# Almond Shell as a Microporous Carbon Source for Sustainable Cathodes in Lithium–Sulfur Batteries

**DOI:** 10.3390/ma11081428

**Published:** 2018-08-14

**Authors:** Almudena Benítez, Marcos González-Tejero, Álvaro Caballero, Julián Morales

**Affiliations:** Dpto. Química Inorgánica e Ingeniería Química, Instituto de Química Fina y Nanoquímica, Universidad de Córdoba, 14071 Córdoba, Spain; q62betoa@uco.es (A.B.); q92gotem@uco.es (M.G.-T.); iq1mopaj@uco.es (J.M.)

**Keywords:** almond, agricultural waste, biomass-derived carbon, sulfur composite, lithium–sulfur batteries

## Abstract

A microporous carbon derived from biomass (almond shells) and activated with phosphoric acid was analysed as a cathodic matrix in Li–S batteries. By studying the parameters of the carbonization process of this biomass residue, certain conditions were determined to obtain a high surface area of carbon (967 m^2^ g^−1^) and high porosity (0.49 cm^3^ g^−1^). This carbon was capable of accommodating up to 60% by weight of sulfur, infiltrated by the disulphide method. The C–S composite released an initial specific capacity of 915 mAh g^−1^ in the Li–S cell at a current density of 100 mA g^−1^ with a high retention capacity of 760 mAh g^−1^ after 100 cycles and a coulombic efficiency close to 100%. The good performance of the composite was also observed under higher current rates (up to 1000 mA g^−1^). The overall electrochemical behaviour of this microporous carbon acting as a sulfur host reinforces the possibility of using biomass residues as sustainable sources of materials for energy storage.

## 1. Introduction

Lithium–sulfur (Li–S) batteries have been considered one of the most promising energy storage systems for several years due to their numerous advantages, such as their high specific energy (2600 Wh kg^−1^) and specific capacity (1675 mAh g^−1^) [1]. These values are much higher than those found in current Li–ion batteries that dominate the market today [2]. The main reason for these differences lies in the number of electrons involved in the electrochemical reaction. Two electrons exchange in Li–S batteries versus one in Li–ion cells. Another advantage of Li–S batteries is the low molecular weight of sulfur. In addition, it is a non-toxic species, environmentally-friendly, very abundant in the Earth’s crust and has a low production cost.

However, there are still a number of drawbacks that have to be overcome in order to carry out the development of these batteries on an industrial scale, and these are the objects of study today. Among the drawbacks of these batteries are (i) the low ionic and electronic conductivity of sulfur (around 5 × 10^−30^ S cm^−1^ at 25 °C) [3]; (ii) the significant volume and structural changes during the reaction due to the density difference between Li_2_S and S (1.66 and 2.07 g cm^−3^, respectively) which causes the pulverization of the material and the loss of electrical contact between the particles [4,5]; and (iii) during the charge/discharge process, long-chain polysulfides are formed which tend to dissolve in the electrolyte, migrate to the Li anode, and cause corrosion (known as the “shuttle effect”) [3,6,7].

The use of activated carbon seems to be a promising solution to these problems by virtue of its properties, namely, its high conductivity and highly developed porous structure which is a fundamental property to be able to accommodate sulfur and improve conductivity. In addition, its structure allows the electrolyte to reach the electroactive sulfur, allowing electrical contact and retaining the polysulfides that are formed during the process. This mitigates its dissolution in the electrolyte and avoids the shuttle effect. There have been a great abundance of carbons studied with different morphologies, origins, and excellent electrochemical properties [8]. However, to obtain these carbonaceous materials, expensive preparation processes or non-sustainable carbon sources are usually necessary.

In this context of sustainability, several researchers have tried to use activated carbon, obtained through simple processes from renewable resources or biomass residues. Some of these carbonaceous materials have been proposed as anodes in Li–ion batteries, as is the case for coals obtained from sugar, cotton wool, peanut, coffee, potato, or banana [9]. Recently, this type of carbon has also been proposed as a positive electrode matrix in Li–S batteries, using starting materials such as rice [10], litchi fruit [11], and mushrooms [12]. Promising electrochemical results have been obtained from this approach. These carbons have the properties sought for cathode matrices, in terms of chemical composition and texture [13].

The use of food sources for energy storage systems has the problem of diverting their use as human and/or animal food, in addition to the need for large tracts of arable land for their production. An example of this problem is the use of corn or sugarcane crops for the production of biodiesels [14]. In this sense, it is worth highlighting the attempts to prepare activated carbon from non-edible components of plant foods, thus taking advantage of waste for a new use and obtaining a cheap starting material. Table 1 summarizes the different investigations carried out to use coal obtained from inedible biomass residues [15,16,17,18,19,20,21,22,23,24,25,26,27,28,29,30,31,32]. Starting from these wastes, carbon of high specific surface area (200–2700 m^2^ g^−1^) and high porosity (0.26–1.50 cm^3^ g^−1^) can be obtained by simple activation and pyrolysis processes. The electrochemical properties of these biomass-derived materials are remarkable, typically showing capacities below 1000 mAh g^−1^. Chulliyote et al. demonstrated the outstanding performance of a sulfur-pinecone derived carbon composite due to the N and O co-doped hierarchical porous carbon layers which enhance the sulfur utilization and mitigate the polysulfide shuttle [25]. A remarkable performance under high rates was found by Chem et al. in coconut-derived carbons with dual micro-mesoporosity [26]. Of special interest are also the results found in carbon derived from biomass with microporous morphology (<2 nm) [17,18,19,22,29]. The micropores may bring about strong interactions between the polysulfides and the pore walls of the carbon matrix due to the overlap of the adsorbate–adsorbent interaction potentials from both sides of the pores [33,34]. The resulting chemical bonds between the polysulfides and the carbon matrices [35,36] also reduce the polysulfide dissolution effectively. The electronic contact of sulfur encapsulated in large mesopores and macropores is quite limited, which results in considerable polarization (it is not favourable for the capacity and rate capability). KOH and ZnCl_2_ were the activating agents used in all carbons described in Table 1, except in reference [15] where H_3_PO_4_ was used. Phosphoric acid offers some advantages with regards to physical activation [37]. As a result, this substance has been used preferably in the activation of a large number of lignocellulosic materials [38], including fruit stones [39]. 

The almond shell (AS) is a non-edible residue of lignocellulosic nature that is generated as a result of the shelling of the almond which is a management expense for the producer of the fruit. So, almond shells are a low-cost, relatively abundant agricultural by-product. Of the total weight of the almond, 25% corresponds to the fruit and 75% to the husk. Previous studies have focused on obtaining active carbons from almond shells by different methodologies [40,41]; however, no studies have been conducted on their use as a material for energy storage systems. Herein, we propose the preparation of a microporous carbon from AS by activation with phosphoric acid as the basis of a composite electrode with sulfur to act as a cathode in Li–S batteries. The resulting ASC/S composite exhibits superior electrochemical properties in terms of capacity, cycling behaviour, and rate capability. It also demonstrates comparable performance with other micro or mesoporous carbons synthetized by more complex and costly processes. To the best knowledge of the authors, there are still no reports about almond shell-derived carbon as a cathode material for Li–S batteries.

## 2. Materials and Methods 

### 2.1. Synthesis of Activated Carbon (ASC) and Sulfur Composite (ASC/S)

Activated carbon (ASC) was synthesized from almond shells that had previously been milled using a ball mill (Restch PM100, Retsch GmbH, Haan, Germany) and sieved after grinding to obtain a fine powder. Phosphoric acid (85%, Sigma-Aldrich, San Luis, CA, USA) was used as the activating agent in an H_3_PO_4_/ASC mass ratio of 1:1; this agent was contacted with the ASC for 3 h at 85 °C. After this process, the obtained product was dried for 24 h at 120 °C and then ground in a mortar. The conditions of the carbonization process were analysed in this study by varying the heating ramp, the target temperature, and the maintenance time at temperature. The calcination process was carried out under a nitrogen atmosphere (flow: 50 mL min^−1^). Afterwards, carbonized ASC was washed with distilled water until the reaction had been neutralized. ASC was finally obtained by drying at 120 °C in an oven (Buchi, Flawil, Switzerland) for 12 h, and it was ground in an agate mortar.

**Scheme 1:**
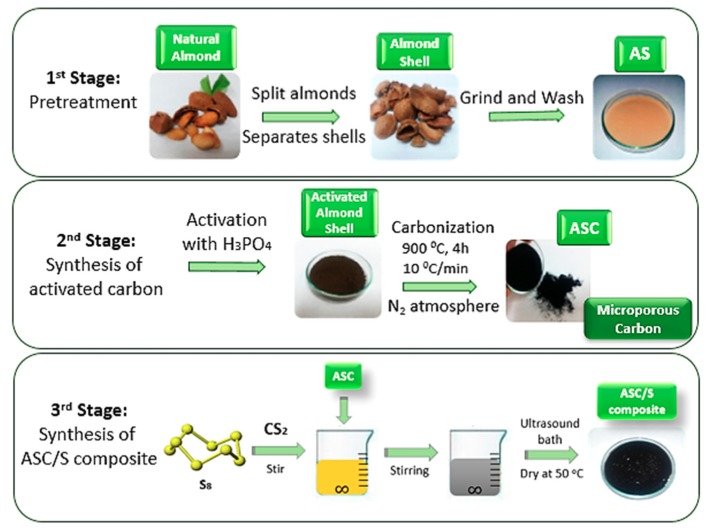
Different steps followed for the preparation of the activated carbon (ASC) and activated carbon/sulfur (ASC/S) composite.

The activated carbon/sulfur composite (ASC/S) was obtained by a process known as the “disulphide method”, based on the dissolution of elemental sulfur in an organic solvent, carbon disulphide and its subsequent mixing with activated carbon. The conditions were as follows: Elemental sulfur (700 mg) was dissolved in carbon disulphide (5 mL) under magnetic stirring for one hour. Then, the ASC (300 mg) was added, and the stirring was maintained for a further 15 min. The resulting suspension was subjected to ultrasound to promote homogeneity until the solvent evaporated. Finally, the obtained ASC/S composite was dried overnight at 80 °C (Scheme 1). 

### 2.2. Carbon and Composite Characterization

The sulfur content and the thermal properties of the samples were determined by thermogravimetric analysis (TGA) with a Mettler Toledo TGA/DSC (Mettler-Toledo, Columbus, OH, USA) under a nitrogen or oxygen atmosphere, heating the samples from 25 to 800 °C at 5 °C min^−1^. Specific surface areas were examined with a Autosorb iQ/ASiQwin (Quantachrome Instruments, Boynton Beach, FL, USA), using nitrogen as the adsorbent. The pore size distributions of the carbon in the micropore and narrow mesopore ranges were obtained by applying the density functional theory (DFT) method. The structural properties of carbon and composite were examined with a Bruker D8 Discover X-ray diffractometer (XRD, Bruker, Billerica, MA, USA). The patterns were acquired between 10° and 80° (2θ), with a step size of 0.04° and a time of 1.05 s per step. Raman measurements were carried out with a Renishaw inVida microscope equipped with a detector Renishaw charge-coupled device CCD Camera (578 × 400) (Renishaw, Wotton-under-Edge, UK) and a laser of 532 nm edge in line focus mode. Sample morphology was examined with a Jeol JSM 6300 scanning electron microscope (SEM, Jeol, Tokyo, Japan). The SEM was coupled to a microanalysis system (LECO CHNS-932) and measured by energy dispersive X-ray spectroscopy (EDS, Jeol, Tokyo, Japan). 

### 2.3. Cathode Preparation and Electrochemical Measurements

The cathode was prepared by mixing the activated carbon/sulfur composite (ASC/S) with a Super P carbon black (conducting agent, Timcal, Paris, France) and polyvinylidenedifluoride (PVDF, binder, Sigma-Aldrich, San Luis, CA, USA) in a mass ratio of 80:10:10, in a *N*-methyl-2-pyrrolidone (NMP, Sigma-Aldrich, San Luis, CA, USA) solution. The slurry was deposited on Al foil by doctor blade deposition. The electrodes were cut in 13 mm diameter disks with a sulfur loading of 2.0 mg cm^−2^ and then were dried at 45 °C under vacuum for 3 h. 

CR2032 coin-cells assembled inside a glove box (Ar-filled, M-Braun 150, M-Braun, Garching, Germany) were used for electrochemical measurements. Li metal foil served as the counter and reference electrodes. The electrolyte was LiTFSI 1.0 M (Sigma-Aldrich) and LiNO_3_ 0.4 M in 1,3-dioxolane (DOL, Sigma-Aldrich) and 1,2 dimethoxyethane (DME, Sigma-Aldrich) (1:1 *v*/*v*) soaked in a polyethylene membrane (Celgard, Charlotte, NC, USA) as a separator. A 0.4 M concentration of the LiNO_3_ additive was used in the electrolyte to prevent lithium dendrite growth and to avoid parasitic reactions [42,43]. The cyclic voltammetry (CV) curves were recorded with an Autolab PGSTAT–204 (Metrohm, Herisau, Switzerland) at a scan rate of 0.05 mV s^−1^ within the 1.7–2.7 V range. Cycling tests were performed on an Arbin BT2143 (Arbin Instruments, College Station, USA) potentiostat–galvanostat system within the potential window of 1.7–2.7 V at various current rates. Both the specific capacity and current rate referred to the sulfur mass in the positive electrode (48 wt %). 

## 3. Results and Discussion

### 3.1. Structural and Textural Properties

**Figure 1:**
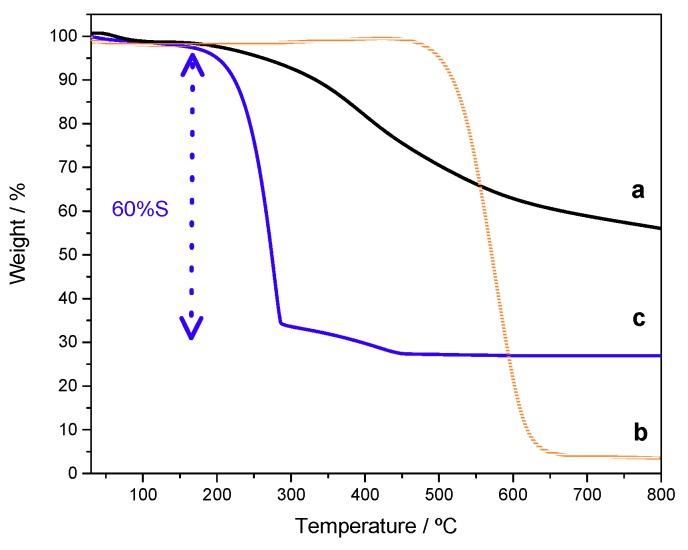
Thermogravimetric (TG) analysis of (**a**) AS [N_2_], (**b**) ASC [O_2_] and (**c**) ASC/S composite [N_2_].

Almond shells are mainly composed of cellulose and hemicellulose. Thus, the carbon yield from almond shells is expected to be significantly high, along with advantages such as a higher surface area and porosity due to its cellulose content. In order to demonstrate the viability of almond shells as a sustainable source of carbon, an elemental analysis of milled, washed, and dried shells was carried out, showing a carbon content of 48.4% by weight. The thermogravimetric analysis of this material in an inert atmosphere (Figure 1a) showed a significant and continuous loss of mass below 600 °C, which indicates that the pyrolytic process occurs below this temperature and in several stages. These stages are associated with the processes of degradation of the cellulosic components of the almond shell.

**Figure 2:**
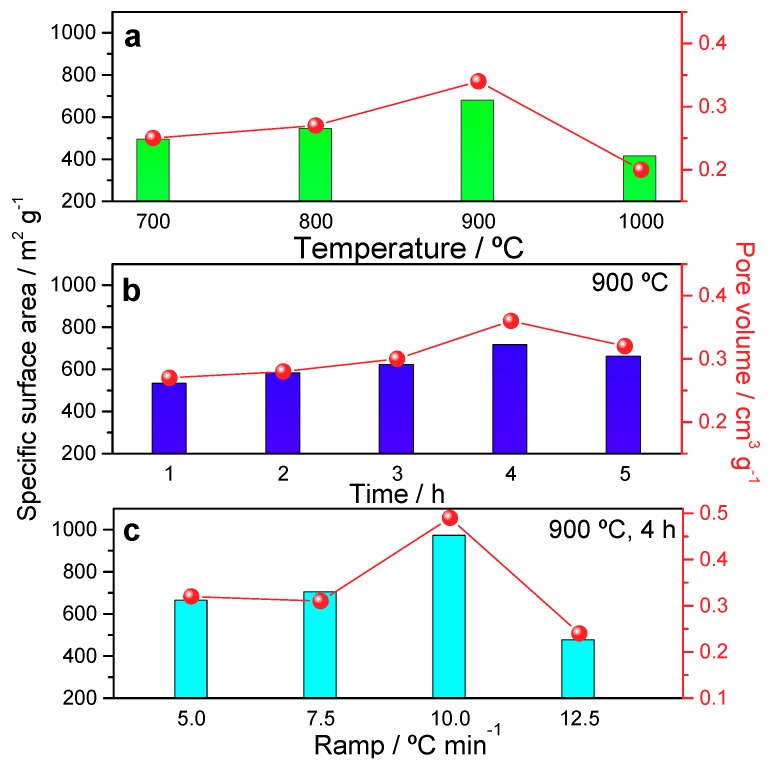
Effects of carbonization parameters on the textural properties of the almond shell-derived carbons: (**a**) temperature, (**b**) time, and (**c**) temperature ramp.

Once the almond shell had been activated with phosphoric acid, several parameters of the carbonization process were analysed in order to obtain an active carbon with the highest possible values of specific surface area and pore volume. Both characteristics are decisive in the performance of active carbon as a matrix of sulfur in an electrode. The effects of the temperature, carbonization time, and temperature ramp were determined. In Figure 2, the BET (Brunauer, Emmett and Teller) surface values and total pore volume according to the three described parameters are shown. When the effect of the target temperature was analysed, keeping the time (5 h) and the ramp rate (5 °C min^−1^) fixed, it was verified that the optimum values were reached at 900 °C (Figure 2a). During pyrolysis, the complete volatilization of the hemicellulose, cellulose, and the lignin components of the almond residue caused the opening of pores, enabling the development of the porosity. This development of porosity increased until it reached its maximum at 900 °C. However, when the temperature increased to 1000 °C, a loss in surface properties was observed due to the shrinkage of the pores and the re-alignment of the carbonaceous structure [44]. The effect of the pyrolysis time was analysed by maintaining this calcination temperature (Figure 2b). The increase in calcination time had a positive effect on both the porosity and the surface. However, times longer than 4 h were associated with negative effects on the surface which may be due to a widening of the micropores [45]. Additionally, the influence of the temperature ramp was analysed (Figure 2c). The most promising active surface and porosity values were obtained with a rate of 10 °C min^−1^.

**Figure 3:**
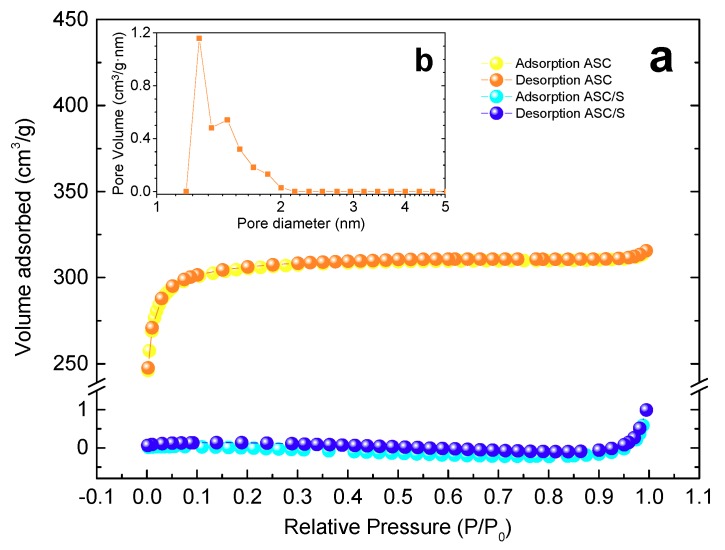
(**a**) Adsorption isotherm of nitrogen at 77 K for ASC and ASC/S composite; (**b**) density functional theory (DFT) plot for ASC is shown in the inset.

The results show that the temperature of 900 °C, calcination time of 4 h, and ramp of 10 °C min^−1^ were the most favourable conditions to obtain an almond-derived carbon with suitable properties to act as a sulfur host. The N_2_ adsorption isotherm analysis of the carbonized sample under these conditions (ASC) is shown in Figure 3a and demonstrates the microporous nature of the carbon. The shape of the isotherm is associated with Type I of the IUPAC classification, where the adsorption occurs at low relative pressures, typical of microporous solids, presenting a surface area of 967 m^2^ g^−1^. Likewise, the analysis of the pore size by means of the DFT method (Figure 3b) confirmed the pore size range of 1.5–1.8 nm. The volume of carbon micropores calculated by a t-plot was 0.44 cm^3^ g^−1^, compared to the V_total_ which was determined to be 0.49 cm^3^ g^−1^. In other words, the microporosity encompassed 90% of the complete porosity of the ASC. These results are in the order of those collected for the biomass-derived carbon in Table 1, even considering that practically all of them were activated by different agents (mainly KOH). The textural properties of the ASC confirmed the tendency to form microporous carbon from biomass by activation with phosphoric acid, which was previously reported by Olivares-Marin et al. [46]. Microporous structures can be developed by carbonization at high temperatures between 700 and 900 °C, by creating narrow micropores with dilute H_3_PO_4_ solution in the impregnation of a biomass. Previous studies of almond shells also confirmed the ease of this biomass waste to form microporous activated carbon [40,41]. The BET surface and pore volume values slightly surpassed those reported in these works, where activation was carried out by means of carbon dioxide or ammonia gas stream, confirming the viability of phosphoric acid as an activating agent for this biomass residue.

The chemical composition of the ASC was determined by EA (elemental analysis), TGA, XRD, and Raman spectroscopy. The elemental analysis revealed a C content higher than 90% which was confirmed by TG in oxygen (Figure 1b). A weight loss of 95%, associated with the combustion of coal between 500 and 600 °C, was observed in the thermogram. According to the data presented in Figure 1b, the carbon obtained by chemical activation of almond shells by phosphoric acid has a low ash content. This is most likely a consequence of a low content of inorganic substances in the precursor and the fact that a considerable part of the mineral substance could have been removed as a result of reaction with H_3_PO_4_, or could have been washed out in the washing process after carbonization. 

**Figure 4:**
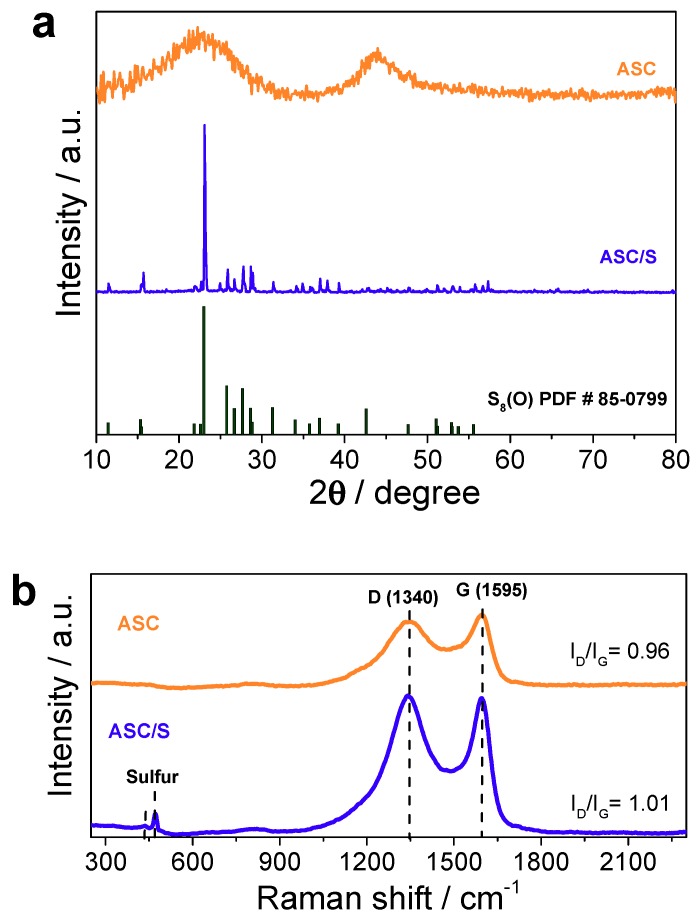
(**a**) XRD pattern and (**b**) Raman spectra of ASC and ASC/S composite.

The XRD spectrum of the ASC (Figure 4a) only showed the two broad peaks typical of highly disordered carbons, located at 25° and 45°. Both low intensity peaks were indexed as the (002) and (100) crystallographic planes of graphite. No additional peaks were detected from crystalline impurities. The XRD spectrum of the ASC/S composite showed a series of well-defined peaks, indicating high crystallinity, corresponding to polymorph orthorhombic sulfur (PDF # 85-0799). The high sulfur content blurred the detection of the carbon by XRD. 

When analysing the ASC/S composite isotherm (Figure 3), it was verified that the adsorption properties decreased drastically, decreasing the value of the surface area below 1 m^2^ g^−1^. The shape of the N_2_ adsorption isotherm changed to Type II, as is typical of non-porous materials. These data confirm that the sulfur completely occupied the pore system of the carbonaceous matrix. The sulfur content of the ASC/S composite can be correlated with the pore volume with Equation (1):

(1) WS (%)V=(ρS·V)(ρS·V+1)×100,

where *V* is the total pore volume of the carbon host, and *ρS* is the density of elemental *S* (~2.07 g cm^−3^) [12]. The theoretical amount of sulfur that the synthesized ASC can hold when applying the above formula is 55% by mass. However, it was observed experimentally by TGA that the amount rises slightly up to 60% (see Figure 1c). This is because part of the sulfur can be deposited on the surface of the activated carbon, in addition to the pores. The XRD pattern of the bulk composite confirmed this high sulfur content by well-defined peaks assigned to the orthorhombic sulfur polymorph (Figure 4a).

The structures of the ASC and ASC/C composites were further confirmed by Raman spectroscopy, as shown in Figure 4b. There were two bands at around 1347 and 1597 cm^−1^ for the ASC sample. The Raman spectra showed a typical curve of partially graphitized carbon with the well-known D-band and G-band, which indicated good electrical conductivity. Generally, the D band indicates disordered graphitic carbon, while the G band is ascribed to the crystalline graphite structure. The relative intensity ratio of I_D_/I_G_ is proportional to the defect degree of carbon materials. The value of I_D_/I_G_ for ASC was 0.96, indicating a higher electronic conductivity [47]. The I_D_/I_G_ reached 1.01 after impregnation with 60 wt % sulfur, implying that more lattice defects emerged [48]. Crystalline sulfur exhibits characteristic peaks at 430 and 470 cm^−1^ [49]. The low intensity sulfur peaks observed in ASC/C composite indicated that sulfur is incorporated into the carbon matrix successfully by the “disulphide method”. 

**Figure 5:**
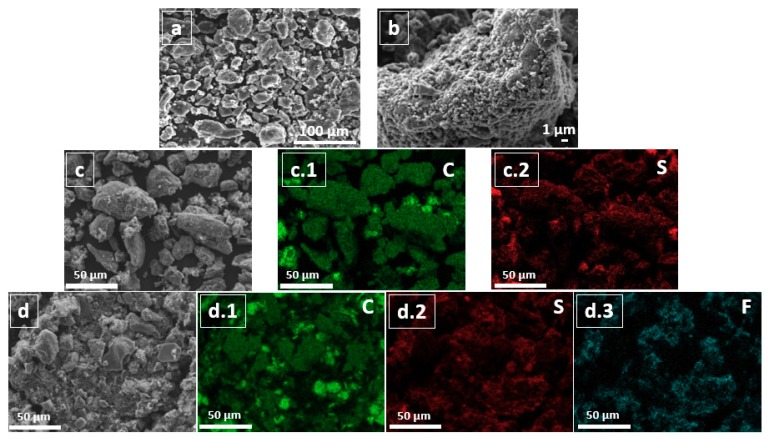
SEM images of (**a,b**) ASC, (**c**) ASC/S composite with C (**c.1**) and S (**c.2**) elemental mapping, and (**d**) ASC/S electrode with C (**d.1**), S (**d.2**), and F (**d.3**) elemental mapping.

The SEM images (Figure 5a,b) revealed a complex morphology for the carbon microparticles (average size of less than 50 microns), indicating a tendency to be aggregated with a smooth surface. After sulfur loading, the observed invariant morphology of the ASC particles and the absence of sulfur agglomerates on its surface indicated a uniform distribution and better confinement of sulfur, as shown in Figure 5c. Figure 5c also shows the elemental maps of carbon and sulfur for the ASC/S composite and confirms that the electroactive element was homogeneously distributed in the framework of the porous carbon. The “disulphide method” allowed sulfur to be fully infiltrated and homogenously distributed on the microporous carbon, as reported for previous disordered carbon [50]. The homogeneous dispersion of the sulfur in the ASC/S composite is corroborated by observing the mapping images of Figure 5d. The presence of fluorine due to the binder (PVDF) along the electrode was also detected.

### 3.2. Electrochemical Properties

**Figure 6:**
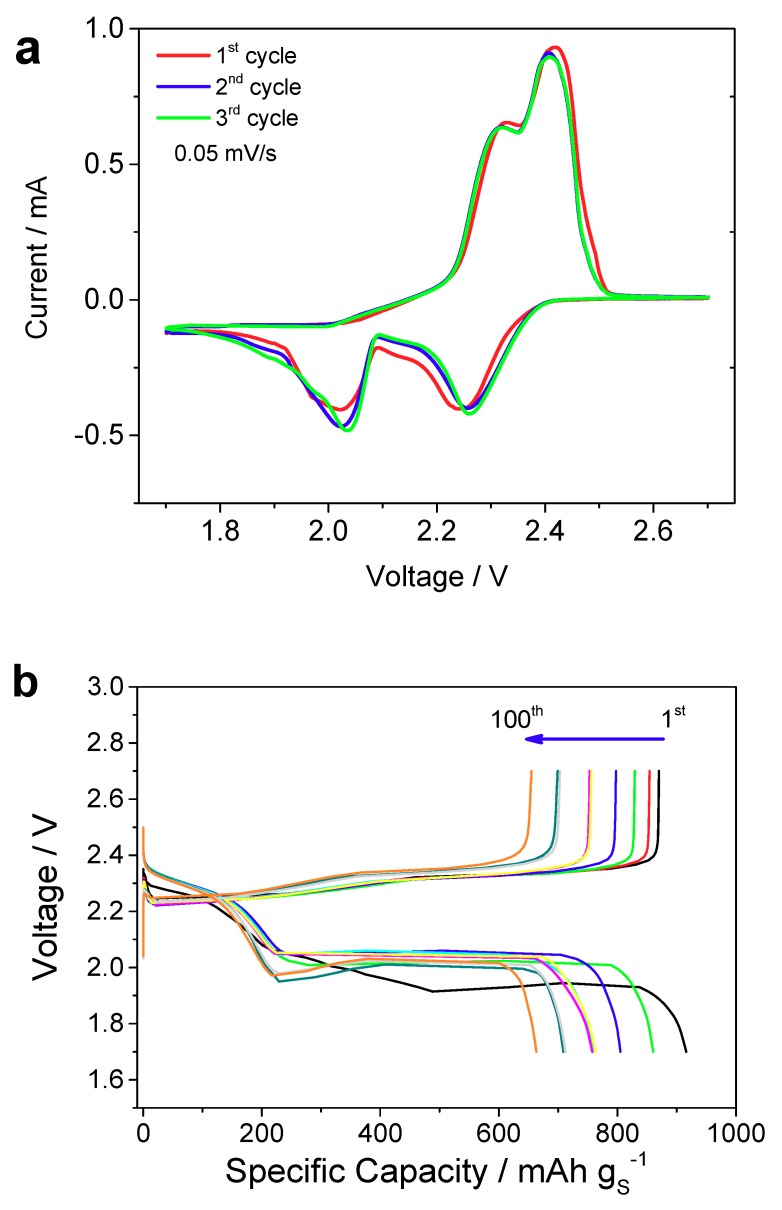
(**a**) Cyclic voltammetry (CV) curves of ASC/S composite measured in the potential window of 1.7–2.7 V at a scan rate of 0.05 mV s^−1^. (**b**) Discharge/charge curves of ASC/S composite recorded at a current of 100 mA g^−1^.

The electrochemical performance of ASC/S cathodes in Li–S cells was evaluated using cyclic voltammetry (CV) and cycling response under constant current. The CV curves recorded at a scanning rate of 0.05 mV s^−1^ in the range of 1.7 to 2.7 V are shown in Figure 6a. In the first cathodic scan, two reduction peaks centered at 2.26 and 2.02 V are well defined and can be assigned to the multistep reduction process of sulfur [51]. The first peak is generally explained by Equation (2) and is attributed to the opening of the S_8_ ring and the consecutive formation of long-chain polysulphides. These species are transformed to short-chain polysulfides according to Equation (3), and stage collected in the second cathodic signal:

(2) 2 Li+nS→Li2Sn (4≤n≤8),

(3) 2 (n−1) Li+Li2Sn →n Li2S,

According to the anodic scan, the oxidation process of the ASC/S composite in the Li–S cell also occurred in two stages. This profile can be defined as a wide peak centered at ca. 2.35 V. The asymmetric signal is ascribed to the reverse reactions described in the cathodic scan. The asymmetry of the peak is caused by the slow kinetics of these reactions, leading to polarization and overlapping of the signals of both reactions. In subsequent cycles, the shapes of the curves were not modified, demonstrating that the described electrochemical processes are highly reversible.

To further investigate the electrochemical performance of ASC/S composite, galvanostatic cycling performance experiments were carried out in coin cells with Li metal as the anode. All the capacity values referred to the mass of sulfur. Figure 6b shows the discharge/charge profiles of the composite electrode between 1.7 and 2.7 V for different cycles. The main characteristic of the first discharge curve was the presence of two voltage plateaus ascribed to the two-step reaction of S with Li during the discharge process. On charging the cell, a strong polarization was observed, and the plateaus associated to the two reduction steps were poorly defined and overlapped, which is consistent with the wide and asymmetric peak that described the oxidation process in the CV (Figure 6a). 

**Figure 7:**
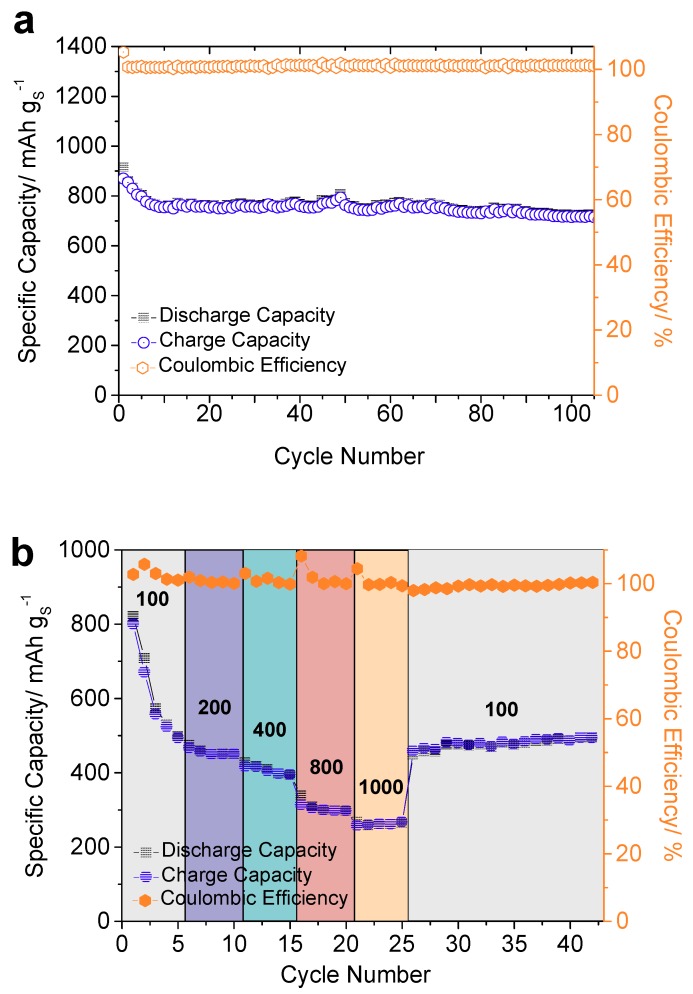
(**a**) Cycling performance of the ASC/S composite at a current of 100 mA g^−1^ and (**b**) rate capability test at various current densities.

The cycling properties of the ASC/S composite in Li–S cells at a rate of 100 mA g^−1^ are shown in Figure 7a. The discharge capacity values varied between 916, 860 and 760 mAh g^−1^ for the first, second and hundredth cycles, respectively. As is typical of Li–S batteries, the composite electrode did not reach the values of the theoretical capacity; however, this deficiency was counterbalanced by high stability during prolonged cycling and the excellent efficiency of the cell. The decrease in specific capacity was observed as a smooth and gradual process, losing only 1.56 mAh g^−1^ per cycle (barely 0.90 mAh g^−1^ per cycle if the first activation cycle is ignored). Additionally, the coulombic efficiency remained at around 99% after the first few cycles. The improved capacity retention in this work was due to the microporous structure that acted as a reservoir, retained the polysulfides, and inhibited the shuttle effect.

The rate capability of the cell with the ASC/C composite electrode is shown in Figure 7b, which reveals reversible capacities of 450, 400, 300, and 280 mAh g^−1^ at current rates increasing from 200 to 400, 800, and 1000 mA g^−1^. A coulombic efficiency higher than 99% was also present. The remarkable stability of the ASC/S composite electrode is suggested in Figure 7b, with the recovery of 94% of the initial capacity by decreasing the current to 100 mA g^−1^ at the 25th cycle. The results indicate that the ASC/S composite can tolerate high current charge/discharge cycling, thus demonstrating a high rate capability and great reversibility.

The electrochemical performance of the ASC was in the order of microporous biomass-derived carbons obtained by activation with potash, such as pomelo peels [17] or wheat straw [18] and improved upon those reported for corncob [19]. The improved capacity retention in this work was due to the microporous structure in ASC, which can effectively adsorb the polysulfides and suppress the shuttle effect, improving the cycling performance of ASC/S composite. Thus, the activation of biomass-derived carbons by phosphoric acid for Li–S batteries is an affordable method to obtain sustainable microporous carbon with a high energy performance. Frequently, remarkable electrochemical performances are reported using high-cost carbon matrices, such as carbon nanotubes or graphene aerogel, or complicated scaling processes, such as chemical vapor deposition (CVD) [52,53,54,55,56]. The electrodes based on this activated carbon require less complex processing methods than those reported for other high-performance cathodes in Li–S batteries, demonstrating a clear advantage in terms of cost and scalability. 

## 4. Conclusions

Biomass-derived carbons have been widely studied as potential candidates to solve two of the main problems of lithium–sulfur battery technology: the poor electronic conductivity of elemental sulfur and the shuttle effect induced by the solubility of the polysulfides in the electrolyte. In particular, the search should focus on carbons derived from inedible biomass. In addition to the sustainable nature of this resource, the synthesis procedures required are less complex than those for carbonaceous materials prepared from carbon-based chemicals. Carbon derived from almond shells and activated with phosphoric acid possess the required textural properties for this application, namely, a high surface area for sulfur hosting and a microporous system that traps polysulfides and hinders their solubility in the electrolyte during the charge–discharge processes. The electrochemical performance of the ASC/S composite acting as the cathode of the Li–S battery fully confirmed this expectation. High specific capacity values were delivered by the batteries when were cycled under both low and high current rates. The good stability in the extended cycling together with the excellent coulombic efficiency allows us to conclude that this biomass-derived carbon could be a sustainable source of electrodes for Li–S batteries. The low cost and easy scaling of the processing for this activated carbon are clear advantages over other sophisticated electrode preparation methods for Li–S batteries. 

## Figures and Tables

**Table 1:** The use of active carbon from non-edible biomass residues as cathodes in Li–S batteries.

Carbon Source	S_BET_ (m^2^ g^−1^)	V_pore_ (cm^3^ g^−1^)	Specific Capacity ^[a]^/Rate ^[b]^	Ref.
Cherry pits	1662	0.97	915/100	[15]
			700/837	
Olive stone	587	0.33	670/100	[16]
Pomelo peels	1533	0.83	760/335	[17]
Wheat straw	1066	0.62	920/167	[18]
			440/1675	
Corncob	1198	0.67	600/167	[19]
	2724	1.49	720/558	[20]
Corn stalks	140	0.26	750/335	[21]
Coir pith	1952	0.86	600/167	[22]
			470/837	
Soybean residues	2690	1.34	750/334	[23]
			400/1675	
	1232	0.54	450/837	[24]
Pinecone	2065	1.50	1260/167	[25]
Coconut shell	2160	0.68	1030/837	[26]
			1030/1675	
Walnut shell	2318	1.13	910/167	[27]
Banana peel	220	0.76	700/334	[28]
	2741	1.23	750/1675	[29]
Rice husk	1098	-	200/167	[30]
			350/334	
	665	0.31	690/837	[31]
			580/1675	
Mandarin peels	1077	0.57	790/83	[32]
Almond shell	967	0.49	760/100	This work
			400/400	
			300/800	

^[a]^ mAh g^−1^, ^[b]^ mA g^−1^.
